# Additional Glance on the Role of *Dientamoeba fragilis* & *Blastocystis hominis* in Patients with Irritable Bowel Syndrome

**Published:** 2018

**Authors:** Ayman Nabil IBRAHIM, Ayman Mohamed AL-ASHKAR, John Talaat NAZEER

**Affiliations:** Dept. of Medical Parasitology, Faculty of Medicine, Ain-Shams University, Cairo, Egypt

**Keywords:** *Dientamoeba fragilis*, *Blastocystis hominis*, Irritable bowel syndrome

## Abstract

**Background:**

Irritable bowel syndrome (IBS) is a functional gastrointestinal disease with high population prevalence. *Dientamoeba fragilis* and *Blastocystis hominis* are reported worldwide as a cause of human gastrointestinal symptoms. This study evaluated the possible link between this syndrome and the infection with *D. fragilis* and *B. hominis* in Egypt.

**Methods:**

Overall, 310 stool samples (160 from IBS patients and 150 from controls) were obtained from Tropical Medicine Outpatient Clinic, Faculty of Medicine, Ain Shams University, Cairo, Egypt between Mar 2015 and Feb 2016. All the stool specimens underwent direct examination and Trichrome staining. Each sample was cultivated on Jones and Robinson's media.

**Results:**

Overall, 42 cases (28%) showed *B. hominis* and 2 cases (1.3%) for *D. fragilis* infections. After performing the culture methods for *B. hominis* and *D. fragilis,* detections increased to 50 cases (33.3%) and 3 cases (2%), respectively. While among 150 controls 18 (12%) positive samples were detected as *B. hominis.*

**Conclusion:**

There may be a possible relationship between the presentation of irritable bowel syndrome and *D. fragilis* and *B. hominis* infections, which have to be excluded first.

## Introduction

Irritable bowel syndrome (IBS) is a functional gastrointestinal disease manifested by nausea, vomiting, and diarrhea usually is described as small volumes of loose stool preceded by urgency, frequent defecation, diffuse abdominal pain, distention, and alternation in bowel habits in absence of a specific organic pathology. Several global studies recorded the prevalence of IBS to fall between 10%–20% (1). Diagnosis of Irritable Bowel Syndrome relies on the exclusion of other infections or bowel diseases; however, the most important diagnostic factor is the fulfillment of ROME III criteria (2). The therapeutic treatment targets the relief of symptoms with addition of psychotherapy (3).

*Dientamoeba fragilis* is a human intestinal protozoan parasite, commonly reported worldwide and associated with gastrointestinal manifestations (4). Firstly, mistaken by Jepps and Dobell who classified it as an amoeba (5). Then it is a trichomonad without flagella by molecular studies and electron microscopy (6). *Dientamoeba* remains neglected despite many reports of its pathogenic potential. This is due to the lack of studies on *Dientamoeba* compared to other intestinal protozoa such as *Entamoeba histolytica* and *Giardia lamblia*. Therefore, to say that *D. fragilis* is the exact cause of gastrointestinal disturbance, there must be no other pathogen detected (7).

*Blastocystis hominis* is a cosmopolitan human intestinal protozoan parasite (8)*.* It is an anaerobic intestinal parasite of humans and animals. Moreover, *B. hominis* is the most common parasite found in human stool samples (9). Its prevalence rates are variable among countries (10). Prevalence is higher in developing (11, 12) than industrialized (13, 14) countries due to poor personal hygiene and consumption of contaminated food and water (15). The pathogenic ability of *Blastocystis* was widely discussed in different studies to determine whether this parasite was pathogenic or not (16). However, in-vitro and in-vivo studies showed that *Blastocystis* infection might be associated with gastrointestinal symptoms especially in patients with irritable bowel syndrome (17). The most common symptoms associated with *Blastocystis* infection include abdominal pain, diarrhea, and vomiting. There are many reports of patients that showed no other cause of gastrointestinal symptoms other than *Blastocystis* (18). Several studies associated *Blastocystis* with cutaneous manifestations in addition to gastrointestinal symptoms (19).

In Egypt, the two protozoa are prevalent. Prevalence rates ranging from 1% to 30% were recorded for *D. fragilis* (20–23). Rates varying between 1% and 35% were recorded for *B. hominis* (24–28). *B. hominis* was most frequently found in patients with *D. fragilis* (26–29) especially in irritable bowel syndrome patients.

The methods commonly used to diagnose parasitic infections have a poor yield due to lack of experience in stool examination under microscopy, so staining methods and specific techniques such as cultures are needed to improve performance (29).

Newer molecular techniques, real-time PCR (qPCR) with multiplexing targeting the small-subunit (SSU) ribosomal DNA (rDNA), have been developed and very attractive to be used (30). “The implementation of multiplex assays had a tremendous impact on routine parasitology laboratory studies in the developed world. However, the cost of such real-time PCR, assay in developing countries at present would prevent their application to routine diagnosis” (31).

The aim of this work was to evaluate for the first time in Egypt the possible link between patients with irritable bowel syndrome and the infection with *D. fragilis* and *B. hominis.*

## Materials and Methods

This descriptive case-control study in which 310 stool samples were obtained from Tropical Medicine Outpatient Clinic, Faculty of Medicine, Ain Shams University, Cairo, Egypt between Mar 2015 and Feb 2016. The patients were fulfilling ROME III criteria of IBS (160) and the other 150 were controls. The controls were composed of healthy volunteers. The age of the participants ranged between 16–50 yr from both sexes.

These patients underwent thorough history and physical examination. All the stool specimens were processed by fresh direct film microscopy and another examination after staining. Stool culture was done for *B. hominis* and *D. fragilis.*

### Microscopy of fecal smear

Fecal sample microscopy was done as described before (32, 33); Specimens were fixed in both; Polyvinyl alcohol (PVA) and 10% formalin (34) then stained with Ghomori`s Trichrome stain (32) and modified Ziehl Neelsen stain to exclude coccidian infections (35). These preparations were examined under the low power (×10), the high power (×40) and the oil immersion (×100) objectives.

### Culture

For culturing *B. hominis*, Jones medium without starch was used (36). The cultures were incubated at 37 °C and examined after 48 h. If no *B. hominis* were seen up to further 2 d, they were regarded as negative. The sediment was examined under both the low power (×10) and high dry (×40) objectives. Robinson's medium was used to culture *D. fragilis* according to Windsor et al (37).

### Statistical Analysis

The collected data was revised, coded, tabulated and introduced to a PC using SPSS. Data were analyzed using SPSS package ver. 15 (Inc., Chicago, IL). Chi-square test (Fisher’s exact test) was used to examine the relation between qualitative variables. For quantitative data, comparison between two groups was done using Mann-Whitney test (nonparametric *t*-test).

### Ethical consideration

An informed consent was taken from all patients before taking stool samples. The study was approved by the Research Ethics Committee, Faculty of Medicine, Ain Shams University.

## Results

**Fig. 1:**
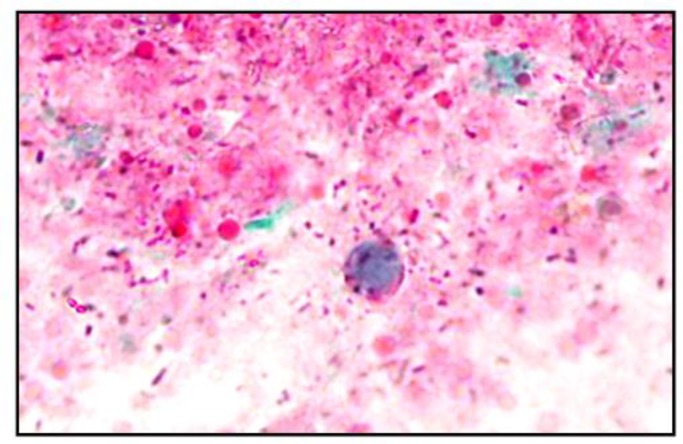
Trichrome stained film (×1000) showing *B. hominis* cyst with rounded vacuolated structure and peripheral nuclei

From the 150 patients presenting with IBS according to ROME III classification, 42 cases (28%) showed *B. hominis* (Fig. 1), 21 cases (14%) showed *Entamoeba coli* and 2 cases (1.3%) for each *D. fragilis* and *Chilomastix mesnili* infections. These results were shown after performing Trichrome staining technique. A note was made of the presence of other parasites such as *Giardia intestinalis* and *Cryptosporidium* spp., and these patients, ten in number, were excluded. No results revealed *Enterobius vermicularis* infection.

However, after performing the culture methods for *B. hominis* and *D. fragilis* detections increased to 50 cases (33.3%) and 3 cases (2%), respectively (Table 1).

**Table 1:** Detection by various tests for *B. hominis* and *D. fragilis* in IBS patients

	***Saline/Iodine No (%)***	***Trichrome No (%)***	***Culture No (%)***
***Blastocystis hominis***			
Positive	42 (28)	42 (28)	50 (33.3)
Negative	108 (72)	108 (72)	100 (66.7)
***Dientamoeba fragilis***			
Positive	0 (0)	2 (1.3)	3 (2)
Negative	150 (100)	148 (98.7)	147 (98)

Comparison between cases and controls regarding *B. hominis* and *D. fragilis* as detected by microscopy and culture methods among cases and controls exhibited high significance in *B. hominis* cases (*P*=0.0001) and non-significant value (*P*=0.08) as regards *D. fragilis* cases*.* Moreover, the detected positive samples for *B. hominis* and *D. fragilis* among the 150 controls are 18 (12%) and zero (0%) respectively (Table 2).

**Table 2:** Comparison between cases and controls regarding *B. hominis* and *D. fragilis* as detected by Microscopy and culture methods

	***IBS (mic/cul)***	***Controls (mic/cul)***	***P-value***
***Blastocystis hominis***			0.0001
Positive	50	18	
Negative	100	132	
***Dientamoeba fragilis***			0.08
Positive	3	0	
Negative	147	150	

* Significant: *P* ≤ 0.05.

** Highly significant: *P* ≤ 0.001.

**Fig. 2:**
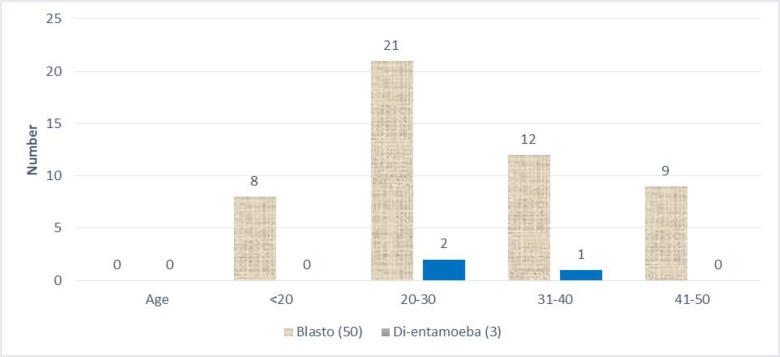
Distribution of *B. hominis* and *D. fragilis* positive cases among their age groups

Distribution of *B. hominis* and *D. fragilis* positive cases among their age groups was shown in Table 3 and Fig. 2. *B. hominis* positive cases were 8 (16%) under age of 20 yr, 21 (42%) from 20 to 30, 12 (24%) from 31 to 40 and 9 (18%) from 40 to 50 yr old. While for the *D. fragilis* positive cases, 2 (66.7%) cases were recorded from 20 to 30 aged group and only one case from 30 to 40 yr old. This may give a correlation of the most affected age group by both parasites which is the 20–30 age groups.

**Table 3:** Distribution of *B. hominis* and *D. fragilis* positive cases among their age groups

***Age(yr)***	***Blastocystis (50)***		***Dientamoeba (3)***	
	N	%	N	%
<20	8	16	0	0
20–30	21	42	2	66.7
31–40	12	24	1	33.3
41–50	9	18	0	0

## Discussion

There is a possible relationship between IBS and *B. hominis* and *D. fragilis* infections especially *B. hominis* infection (33.3%).

This coincides was investigated the prevalence of *Dientamoeba* and *Blastocystis* in irritable bowel syndrome patients (IBS) in Turkey (38). They did not find any stool sample positive for *Dientamoeba* but reported that many patients with IBS were found to have five or more *Blastocystis* parasites per field than control groups and they also reported regression of IBS symptoms after treatment in most of the patients with *Blastocystis* and lastly concluded that there may be a possible link between IBS and *Blastocystis*.

Moreover, *Dientamoeba* in 7.6% of fecal specimens from patients with enteritis was detected (39) and *Dientamoeba* in 0.9% of patients with diarrhea not detected in a control group of 900 patients without bowel complaints (40). Other study detected *Dientamoeba* in 4.5% of patients suffering from non-specific bowel disorders and 2% of patients with diarrhea (41). *Dientamoeba* was detected in 5.4% of patients with bowel complaints using light microscopy in combination with culture, PCR and real-time PCR (23).

Moreover, a study that aimed to screen for *Blastocystis* in colonic stool aspirate samples in patients with and without IBS undergoing colonoscopy and measured the interleukin levels (IL-3, IL-5, and IL-8) (42). They used stool cultures, polymerase chain reaction (PCR) for detection and subtyping of *Blastocystis*. Patients with IBS infected with only *Blastocystis* showed an increase in the interleukin levels which suggested that *Blastocystis* does have an effect on the immune system of the patient.

As an opposite opinion, a study was utilized a population-based sampling frame and Rome criteria assessment to examine the phenomenon of IBS in a “non-sterile” developing population, where repeated exposure to gastrointestinal pathogens is common and found *B. hominis* infection to be more in controls than IBS patients and stated a lack of an association between IBS and parasite infection in the developing nation environment of Nicaragua, Central America (43).

Although molecular methods specially Real-time PCR (qPCR) considered as an attractive technique for laboratory diagnosis of infectious diseases and would give more accurate results with *D. fragilis* (44), this could not be applied in the study due to high expenses and being not a routine method in developing countries.

Consequently, culture method was used as additional and confirmatory technique with the direct and staining as its effectiveness was proved over microscopy (29, 36) and in sometime over the PCR (29).

## Conclusion

There may be a possible relationship between Irritable Bowel Syndrome and *D. fragilis* and *B. hominis* infections, especially *B. hominis* infection.
